# Plasma Levels of Neuron/Glia-Derived Apoptotic Bodies, an In Vivo Biomarker of Apoptosis, Predicts Infarct Growth and Functional Outcome in Patients with Ischemic Stroke

**DOI:** 10.1007/s12975-024-01283-4

**Published:** 2024-08-02

**Authors:** Inmaculada Díaz-Maroto, Beatriz Castro-Robles, Miguel Villar, Jorge García-García, Óscar Ayo-Martín, Gemma Serrano-Heras, Tomás Segura

**Affiliations:** 1 Department of Neurology, General University Hospital of Albacete, Hermanos Falcó, 37, 02008 Albacete, Spain; 2 Research Unit, General University Hospital of Albacete, Laurel, s/n, 02008 Albacete, Spain; 3 Department of Radiology, General University Hospital of Albacete, Albacete, Spain; 4 Instituto de Biomedicina (IB-UCLM), Facultad de Medicina, Universidad de Castilla-La Mancha, Albacete, Spain

**Keywords:** Stroke, Apoptosis, Apoptotic bodies, Biomarker, Infarct growth, Extracellular vesicles

## Abstract

Evidence demonstrating the involvement of apoptosis in the death of the potentially salvageable area (penumbra zone) in patients during stroke remains limited. Our aim was to investigate whether apoptotic processes occur in penumbral brain tissue by analyzing circulating neuron- and glia-derived apoptotic bodies (CNS-ApBs), which are vesicles released into the bloodstream during the late stage of apoptosis. We have also assessed the clinical utility of plasma neuronal and glial apoptotic bodies in predicting early neurological evolution and functional outcome. The study included a total of 71 patients with acute hemispheric ischemic stroke (73 ± 10 years; 30 women). Blood samples were collected from these patients immediately upon arrival at the hospital (within 9 h) and at 24 and 72 h after symptom onset. Subsequently, isolation, quantification, and phenotypic characterization of CNS-ApBs during the first 72 h post-stroke were performed using centrifugation and flow cytometry techniques. We found a correlation between infarct growth and final infarct size with the amount of plasma CNS-ApBs detected in the first 72 h after stroke. In addition, patients with neurological worsening (progressive ischemic stroke) had higher plasma levels of CNS-ApBs at 24 h after symptom onset than those with a stable or improving course. Circulating CNS-ApB concentration was further associated with patients’ functional prognosis. In conclusion, apoptosis may play an important role in the growth of the cerebral infarct area and plasma CNS-ApB quantification could be used as a predictive marker of penumbra death, neurological deterioration, and functional outcome in patients with ischemic stroke.

## Introduction

Despite the successful implementation of recanalization therapies in the acute phase, ischemic stroke remains one of the leading causes of mortality, disability, and dementia worldwide. Moreover, due to the ageing population, the incidence and prevalence of this disease are expected to increase significantly in the coming decades [1–4]. In this epidemiological context, research into treatments in the field of neuroprotection is therefore of particular interest.

In 1977, Astrup and colleagues [5] were the first to describe the existence of two zones within the dependent territory of an occluded cerebral artery. The central zone, or infarct core, suffers irreparable structural damage after the cessation of cerebral blood flow. Surrounding the core is the penumbra zone, tissue at risk but still potentially salvageable, where the reduction in regional cerebral blood flow could trigger the activation of several cellular damage mechanisms (ischemic cascade), ultimately leading to the death of penumbral brain tissue, sometimes even when circulation has been restored [5–7]. This death of the ischemic penumbra in the first hours and even days after cerebral vascular occlusion is the main determinant of infarct size and is therefore the therapeutic target of revascularization therapies. However, early recanalization is not always achieved or is unable to halt the progression of tissue death in the ischemic penumbra in a significant number of infarcted patients, leaving an urgent need for neuroprotective treatment to reduce brain damage after stroke. Several neuroprotective therapies aimed at blocking key points of the ischemic cascade (excitotoxicity, oxidative stress, inflammation, and mitochondrial dysfunction) have been developed. However, after encouraging results in animals, these treatments have failed in humans [8]. An alternative line of research has therefore been proposed, based on the study of the mechanisms responsible for brain cell death in the ischemic penumbra [9–12].

Over the last two decades, several histological and molecular studies in vitro and in vivo using animal models of cerebral ischemia have shown that apoptosis is the main mechanism of neuronal damage in the penumbra [11–24], although other types of regulated cell death, such as autophagy, necroptosis, ferroptosis, parthanatosis, phagoptosis, and pyroptosis, may occur during the ischemic event [16, 25–29]. However, there have been few studies on cerebral apoptosis in patients with ischemic stroke because of limitations in the methods for the detection of this death mechanism, which require tissue samples from patients to be taken invasively or fluorescent or radiolabeled probes to be injected throughout the body [10–30]. Thus, the identification of biomarkers of neuronal and glial apoptosis may represent an important advance in clinical practice by allowing the assessment of processes leading to cell death in stroke patients. In addition, circulating indicators of brain cell death would have the potential to predict the radiological progression of cerebral infarction as well as functional outcome. In this context, our research laboratory developed a novel, rapid, accessible, and reproducible method to monitor human apoptosis in real time. It is based on the non-invasive isolation, quantification, and phenotypic characterization of apoptotic bodies (ApBs), extracellular vesicles (EVs) released during the late stages of programmed cell death [30]. These ApBs have two interesting properties: they contain proteins and genetic material from parental cells, making it possible to identify their origin, and they can cross the blood–brain barrier (BBB) from brain to blood, making it possible to study them in peripheral blood [31]. In this study, we investigated whether apoptosis is involved in the evolution from penumbra to infarct tissue in patients with ischemic stroke. To this end, we measured circulating neuron- and glia-derived apoptotic bodies (CNS-ApBs) in blood samples from patients with ischemic stroke during the first 72 h after symptom onset and investigated their association with infarct growth. Additionally, the clinical utility of plasma CNS-ApBs levels as in vivo marker of early neurological and functional outcomes at 3 months was evaluated.

## Materials and Methods

### Patient Selection and Clinical Protocol

We designed a prospective observational prevalence and association study, recruiting a consecutive cohort of patients with non-lacunar supratentorial ischemic stroke of less than 9-h duration, treated at the General University Hospital of Albacete (Spain) over a 2-year period. We included patients older than 18 years, with functional independence (modified Rankin Scale (mRS) score equal to or less than 2), with brain tissue in a potentially salvageable state (ischemic penumbra), as demonstrated by a brain computed tomography (CT) perfusion protocol [13].

In a total of 71 patients, the following demographic and clinical variables were collected age, sex, toxic habits, vascular risk factors (VRF), history of stroke, ischemic heart disease and/or peripheral arterial disease, usual treatment, initial National Institute of Health Stroke Scale (NIHSS) score, stroke location and etiology (SSS-TOAST classification), presence of stenosis/large vessel occlusion estimated by transcranial Doppler, administration of intravenous thrombolytic therapy, arterial recanalization at 24 h, early neurological course according to changes in NIHSS score over the first 96 h (classified as progressive if the NIHSS score increases by at least 4 points, regressive if it decreases by 4 points or more, and otherwise stable), hemorrhagic transformation, and functional status at 3 months according to mRS (modified Rankin Score) (Table 1). Patients with functional independence were defined as mRS ≤ 2 at 3 months. We also included 8 healthy volunteers matched for age and sex. This study was conducted with the informed consent of the patients according to the Declaration of Helsinki, and the experimental protocol was approved by the Human Ethics Committee of the General University Hospital of Albacete (committee reference: 04/12).

**Table 1:** Main demographics and clinical characteristics of patients on admission

	Stroke patients
Age, year	73 ± 10
Female, %	30 (42%)
Vascular risk factors (%)	
Smoking habit	6 (8%)
Diabetes mellitus	13 (18%)
Hypertension	52 (73%)
Dyslipidemia	33 (46%)
Previous stroke or TIA	11 (16%)
Clinical characteristics	
NIHSS baseline	11 ± 8
Stroke localization	
MCA	61 (86%)
ACA	1 (1%)
PCA	9 (13%)
Stroke treatment	
IV thrombolysis	46 (65%)
Mechanical thrombectomy	2 (3%)
Stroke subtype (%)	
TOAST classification	
Cardioembolic	36 (51%)
Atherothrombotic	3 (5%)
Undetermined	30 (42%)
Others	2 (2%)

Variables are expressed as mean ± SD or as a percentage, as appropriate. TIA transient ischemic attack; NIHSS National Institutes of Health Stroke Scale; MCA medial cerebral artery; ACA anterior cerebral artery; PCA posterior cerebral artery

### Imaging Protocol and Analysis

After enrolment, an early helical CT perfusion scan (multidetector CT, 64 detectors, Phillips Brilliance 64, acquisition of 8 contiguous 5 mm slices; 4 cm along the craniocaudal axis) focused on the middle cerebral artery territory was performed within the first 9 h after stroke onset to semi-automatically determine penumbra and infarct core volumes. The results were analyzed in an Extended Brilliance Workspace, Version 3.5 Brain CT Perfusion Package (Phillips Healthcare). Infarct core and penumbra volumes were calculated using the thresholds proposed by Soares et al. [32]. The penumbra was defined by a mean transit time (MTT) value > 7 s or 145% with respect to the contralateral healthy hemisphere, and the infarct core was defined by a cerebral blood volume (CBV) < 2 ml/100 g.

To determine the final infarct volume, patients underwent a single CT scan 4–7 days after the stroke. The growth of the cerebral infarct (at the expense of recruitment or death of the penumbra) was then measured manually in the same brain region where the ischemic penumbra had previously been calculated using CT perfusion data, using the maximum diameter formula (A*B*C/2). Neuroimages from both CT perfusion and single CT studies (representative images are shown in Fig. 3a) were evaluated by an experienced radiologist blinded to all clinical and analytical variables. For data analysis, the volume of recruited penumbra [initial penumbra volume-infarct growth (final infarct volume-infarct core volume)], initially a quantitative variable, was categorized using an infarct threshold of 20%. Thus, penumbra failure was defined as death of more than 20% of penumbra tissue.

### Blood Sampling and Procedure for Isolating Plasma Apoptotic Bodies

Blood samples (20 ml) were collected in tubes containing 3.2% sodium citrate as anticoagulant at baseline (less than 9 h) and 24 ± 4 h and 72 ± 6 h after the onset of ischemic stroke. Blood samples were then subjected to preliminary low-speed centrifugation (160 × *g* for 10 min at room temperature) within 2 h of collection to separate plasma from blood cells. Apoptotic bodies in plasma were subsequently isolated using a reproducible centrifugation-based protocol developed by our group [30]. Briefly, cell-free plasma was first centrifuged (700 × *g* for 10 min at room temperature) to remove any cells, cellular debris, and large protein aggregates. The supernatant was then subjected to higher speed centrifugation (14,000 × *g* for 30 min at 12 °C) to sediment the circulating apoptotic bodies. As demonstrated in our previous work, by microscopic examination, dynamic light scattering analysis, and proteomic characterization, circulating vesicles isolated from patients with stroke according to the above protocol showed the morphology, size, and protein composition corresponding to apoptotic bodies [30]. The pellet containing apoptotic bodies was resuspended in 0.22 µm filtered TBS buffer and stored at 4 °C for further characterization.

### Quantification and Phenotypic Characterization of Apoptotic Bodies

**Fig. 1:**
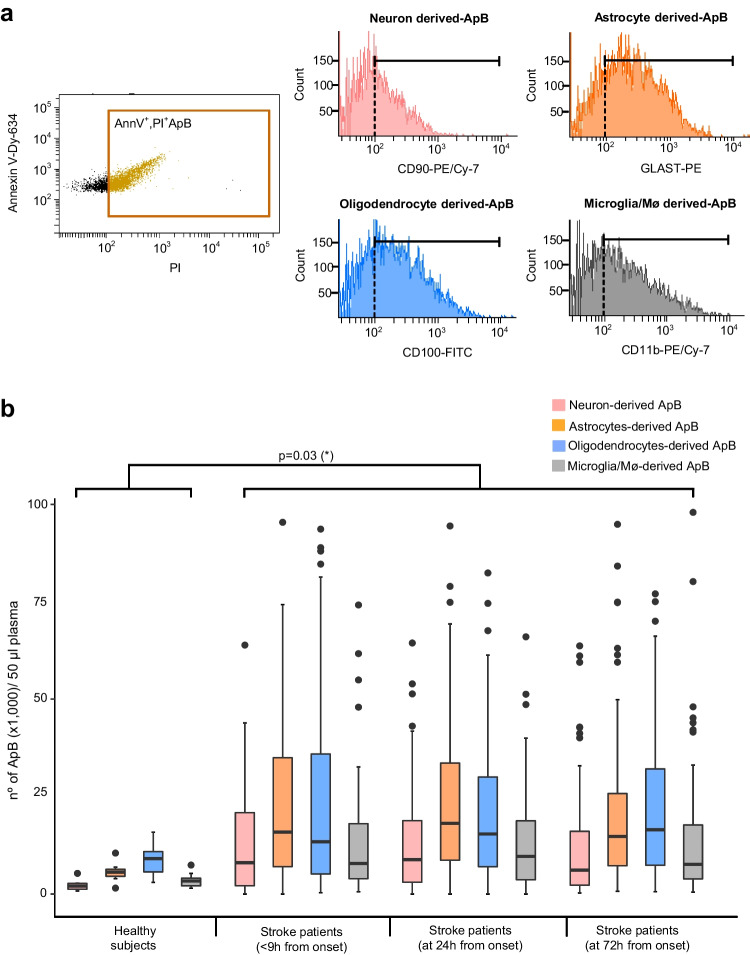
Central nervous system cell-derived apoptotic bodies (CNS-ApBs) in stroke patients and controls. **a** Representative flow cytometry dot plot of annexin V-Dy634 (AnnV)/propidium iodide (PI) double stained plasma apoptotic bodies (ApBs) (left). Flow cytometry histograms showing the expression of neuronal (CD90), astrocytic (GLAST), oligodendrocytic (CD100), and microglial and macrophages (CD11b) markers on AnnV^+^/PI^+^ labeled apoptotic bodies (right). **b** Median concentration of plasma apoptotic bodies derived from neurons, astrocytes, oligodendrocytes, and microglia/macrophages (nº CNS-ApBs/50 µl) in healthy subjects and stroke patients in the first 72 h of symptom onset. The lines in the boxes and the error bars represent the median and 95% confidence interval (CI), respectively

After purification, we used a direct and multi-parameter flow cytometry technique to detect and quantify circulating ApBs isolated from patients with ischemic stroke (*n* = 71) and healthy volunteers (*n* = 8). Since the membrane of these apoptotic vesicles is characterized by the presence of both phosphatidylserine and pores on the external surface, staining with 10 µl of Dy-634 fluorophore-coupled annexin V (AnnV^+^) (Immunostep S.L, Spain), a protein with high affinity for phosphatidylserine, combined with 40 µl of propidium iodide (PI^+^) (10 mg/ml, Invitrogen), a DNA/RNA intercalating agent, was performed to enumerate apoptotic bodies present in the samples from each patient and healthy subject [30]. The concentration of ApBs was therefore determined as the double positive events for annexin V and propidium iodide (AnnV^+^, PI^+^) recorded in the upper right area of a representative flow cytometry dot plot (Fig. 1a, left part) by the volume of the preparation analyzed (50 μl). Next, the determination of cell origin or phenotypic characterization of purified apoptotic bodies was additionally established by flow cytometry using antibodies that specifically recognize neurons and glial cells (Immunostep S.L, Spain). In particular, four volumes of each apoptotic bodies sample, previously stained with annexin V and propidium, were subsequently and separately labeled with either PE-Cy7-conjugated CD90, a membrane protein mainly expressed in neurons within the nervous system; PE-conjugated GLAST (glutamate transporter), an astrocyte marker; FITC-conjugated CD100, a membrane protein (also called Sema4D) expressed selectively by oligodendrocytes and myelin; or PE-Cy7-conjugated CD11b, a marker of microglia and macrophages Thus, the number of apoptotic bodies derived from neurons and glia (CNS-ApBs) was calculated as the number of positive events for a specific marker (CD90^+^, GLAST^+^, CD100^+^, or CD11b^+^) recorded in a representative flow cytometry histogram (Fig. 1a, right part), within the gate of double positive events for AnnV^+^,PI^+^. Thus, CNS-ApB levels were determined as the number of triple-positive events corresponding to apoptotic bodies derived from neurons (AnnV + , PI + , CD90 +) and glia (AnnV ^+^ , PI ^+^ , GLAST ^+^ /AnnV^ +^ , PI ^+^ , CD100 ^+^ /AnnV^ +^ , PI^ +^ , CD11b^ +^) per volume of the preparation analyzed (50 μl). Measurements were made in duplicate and averaged Fig. 1.

### Statistical Analysis

The Mann–Whitney U test and Kruskal–Wallis test were used to examine the possible statistical association between categorical variables (including penumbra failure, neurological outcome, and functional independence at 3 months) and the continuous variable (plasma levels of CNS-ApBs), as CNS-ApB concentration was not normally distributed. The Wilcoxon signed-rank test as a statistical technique for paired-correlated sample analysis was applied to the comparison of plasma concentration of CNS-ApBs from each patient at different periods, < 9 h, 24 h, and 72 h post-stroke. Spearman correlation analysis was used to investigate the possible association between plasma CNS-ApB concentration and both infarct growth at the expense of ischemic penumbra recruitment/death and the volume of established or final cerebral infarct. Furthermore, logistic regression analyses were performed to analyze the variables independently associated with ischemic penumbra area failure and functional independence at 3 months. Finally, the optimal cut-off (value associated with the maximum value of sensitivity and specificity-1) of plasma CNS-ApB levels for predicting death of more than 20% of penumbra tissue and functional dependence was determined using the receiver operating characteristic (ROC) curve.

## Results

### Patient Cohort and Determination of Plasma Levels of Neuron/Glia-Derived Apoptotic Bodies

The study included a total of 71 patients diagnosed with acute hemispheric ischemic stroke with a mean age of 73 ± 10 years, of whom 30 were women (42%), and 8 healthy volunteers (age 74 ± 8 years, 4 men (50%)). The prevalence of classic vascular risk factors was analyzed and the other clinical variables of interest are shown in Table 1. The proportions regarding the location of the infarct (reaching 86% in the territory of the middle cerebral artery) and etiology (predominance of cardioembolic and indeterminate strokes) are influenced by selection bias, since patients with stroke in the vertebrobasilar territory, relevant carotid occlusions, and lacunar infarcts were excluded. From a radiological point of view, the mean volumes of infarct core, penumbra, and established cerebral infarction at 4 to 7 days were 8.5 ± 12.5 cc, 42.9 ± 34.4 cc, and 22.55 ± 28.5 cc, respectively. The mean infarct growth at the expense of penumbra transformation into infarct tissue was 14.24 ± 19.5 cc (mean percentage of infarcted penumbra: 37.91 ± 50.5) and in 52% of subjects there was failure of this zone, explained above as more than 20% of penumbral tissue becoming infarcted. Hemorrhagic transformation of the infarct occurred in 10% (1 patient IH-1, 3 IH-2, 1 PH-1, and 2 PH-2). Regarding the clinical course, 44 patients (62%) had a regressive stroke, 21 (30%) remained stable, and 6 (8%) had a progressive course. Concerning the functional prognosis, 60% of the patients were independent (mRS ≤ 2) at hospital discharge and 65% at 90 days. The in-hospital mortality rate was 7% and 18% at 3 months.

**Fig. 2:**
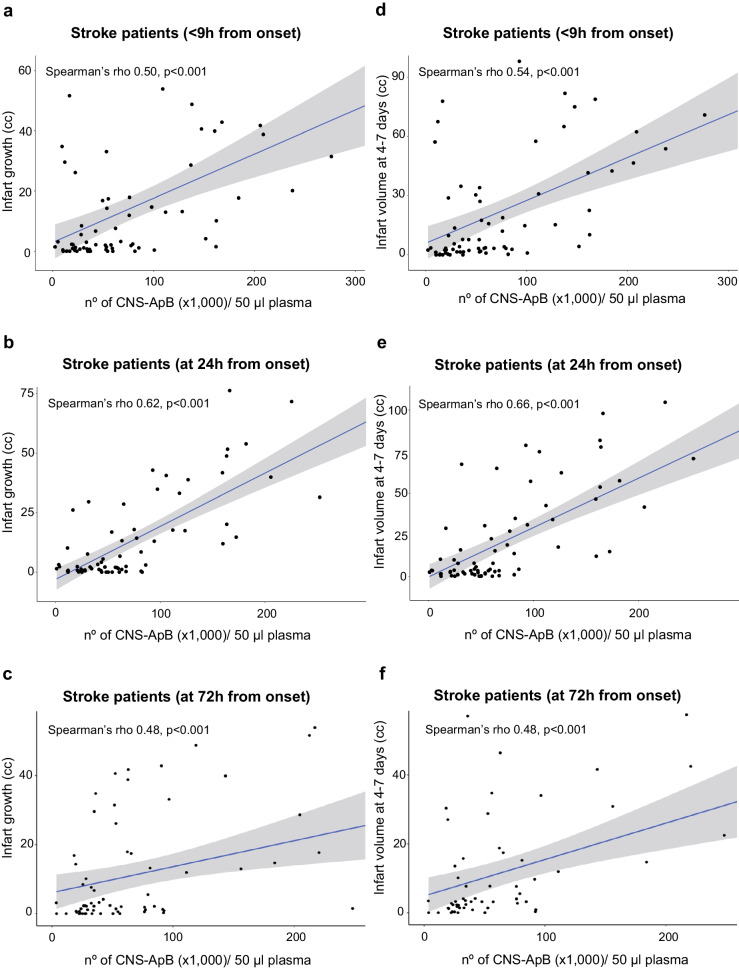
Circulating apoptotic bodies derived from brain tissue cell apoptosis and radiological features in stroke patients. **a**–**c** Positive Spearman’s correlations between plasma concentration of CNS-ApBs at baseline and 24 h and 72 h after stroke and infarct volume growth at the expense of penumbra tissue death and **d**–**f** infarct volume estimated by simple brain CT 4–7 days after stroke onset

We were able to isolate and quantify apoptotic bodies (ApBs), using the reproducible centrifugation-based method combined with flow cytometry analysis developed by our group [30], in the peripheral blood of all patients during the first 72 h of evolution and healthy controls, and we identified those derived from apoptosis of brain tissue cells (neurons, astrocytes, oligodendrocytes) and microglia/infiltrated macrophages (Fig. 1b). The mean levels of circulating neuronal and glial apoptosis-derived ApBs (CNS-ApBs) in stroke patients at the time points analyzed were as follows: the first measurement, obtained less than 9 h after stroke onset (baseline), showed a median of 53,549 CNS-ApBs/50 µl plasma (IQR 23,764–110,172). The medians of the second and third measurements, taken at 24 h and 72 h post-stroke, were 58,721 CNS-ApBs/50 µl plasma (IQR 31,586–105,681) and 52,676 CNS-ApBs/50 µl plasma (IQR 28,000–91,392), respectively. The mean plasma concentration of CNS-ApBs was significantly higher in stroke patients compared to healthy controls at all measurements (*p* < 0.03). Regarding the specific cellular origin of CNS-ApBs, the highest proportions, relative to total neuron/glia-derived apoptotic bodies, came from astrocytes (33.2 ± 1.9% of CNS-ApBs) and oligodendrocytes (29.8 ± 2.4% of total CNS-ApBs), followed by neurons (17.4 ± 1.7% of CNS-ApBs) and microglia/infiltrated macrophages (17 ± 0.7% of CNS-ApBs). These proportions of specific cell lineage ApBs remained stable during the first 72 h (Fig. 1b). It should be noted that plasma concentrations of total CNS-ApBs, as well as brain cell-type specific ApBs, varied across the three stages analyzed after the stroke; however, these differences were not statistically significant. Specifically, circulating levels of neuronal, astrocytic, and microglial/macrophage ApBs reached a maximum peak at the second measurement (24 h after symptom onset), with medians of 8822 ApBs/50 µl plasma (IQR 2919–18,850), 18,072 ApBs/50 µl plasma (IQR 8422–33,800), and 9655 ApBs/50 µl plasma (IQR 3500–18,972), respectively. These levels then decreased to values (6124 neuronal ApBs/50 µl plasma: IQR 2238–16,866; 14,722 astrocytic ApBs/50 µl plasma: IQR 7167–27,647; 7610 microglial/macrophage ApBs/50 µl plasma: IQR 3500–18,972) slightly lower than those observed within the first 9 h after the onset of stroke (8050 neuronal ApBs/50 µl plasma: IQR 1722–22,517; 15,844 astrocytic ApBs/50 µl plasma: IQR 6861–36,189; 7838 microglial/macrophage ApBs/50 µl plasma: IQR 3866–18,500). The plasma concentration of oligodendrocyte-derived ApBs increased similarly to other specific ApBs in the first 24 h (15,344 ApBs/50 µl plasma: IQR 5788–30,438) but remained elevated at 72 h post-stroke (16,444 ApBs/50 µl plasma: IQR 7192–33,47) Fig. 2.

### Plasma Levels of Neuronal and Glial Apoptotic Bodies Are Associated with the Growth of Infarct Area, Neurological Evolution, and Functional Outcome

To assess the clinical utility of neuronal and glial apoptotic bodies (CNS-ApBs) as in vivo markers of activation of apoptotic processes during stroke, Spearman correlation analysis was performed between plasma CNS-ApB levels at the three time points tested after the stroke and infarct expansion. A positive correlation was found between plasma CNS-ApBs levels in all the three measurements and the growth of the infarct area at the expense of penumbral tissue death (Fig. 2a–c), although the strongest relationship was shown by the concentration at 24 h (Fig. 2b) with a Spearman rho of 0.62, indicating a relatively strong positive correlation. The other two determinations (< 9 h and 72 h from onset) were in the moderate correlation range (Fig. 2a, c). We also performed an association analysis between plasma CNS-ApB levels in all the three measurements and final infarct volume at 4–7 days, which showed similar correlations with infarct growth (Fig. 2d–f).

**Fig. 3:**
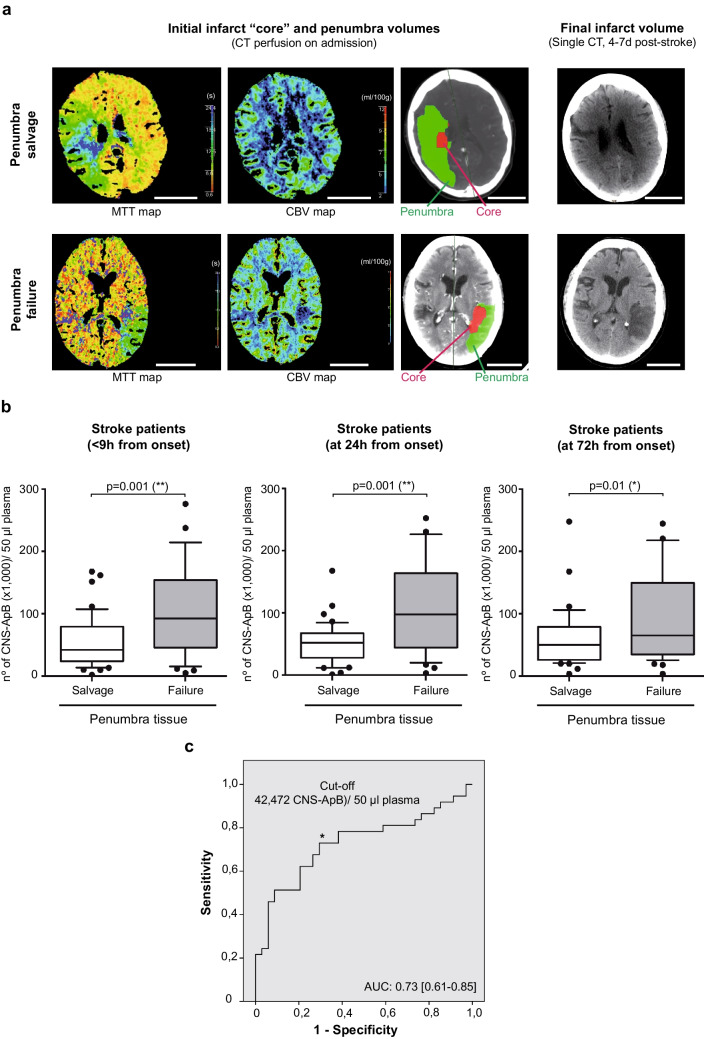
Neuroimaging of brain changes after symptom onset and association between apoptotic bodies derived from neurons/glial cells and penumbral tissue death. **a** Representative images were obtained from CT perfusion scans on admission and from single CT scans performed 4–7 days after stroke in patients who experienced penumbra salvage (those subjects in whom penumbral tissue did not undergo cell death or was recruited in less than 20%) or penumbra failure (recruitment > 20% of tissue). Mean transit time (MTT) and cerebral blood volume (CBV) maps, used to calculate initial infarct (core) and penumbra (potentially salvageable tissue) volumes, respectively, and a brain scan depicting core area (in red) and penumbra area (in green) are shown. Scale bar: 5 cm. **b** Statistical differences (Mann–Whitney *U* test) in plasma CNS-ApB levels between stroke patients with rescued penumbra (salvage) and those with penumbra recruitment (failure). Boxed lines and error bars represent median and 95% CI, respectively. **c** Estimation of the cut-off value of plasma CNS-ApB levels measured in the first 9 h after stroke on ROC curve analysis for predicting penumbra failure

Furthermore, patients with more than 20% penumbra tissue death (penumbra failure), in whom conversion of potentially salvageable cerebral tissue to infarct over time was visualized and calculated using perfusion CT images taken within the first 9 h and a single CT scan at 4–7 days (Fig. 3a), had significantly higher levels of circulating CNS-ApBs at all three measurements (< 9 h, 24 h, and 72 h post-stroke) compared to those with salvaged penumbra (with less than 20% penumbra recruitment) (Fig. 3b), as monitored in the baseline and final radiological studies (Fig. 3a). Notably, in the subgroup of patients with more than 50% penumbra recruitment, there was a statistically significant decrease in the levels of apoptotic bodies from neurons and glia at 72 h post-stroke (median: 70,467 CNS-ApBs/50 µl plasma; IQR 35,415–173,746), following the peak observed within 24 h (median: 159,400 CNS-ApBs/50 µl plasma; IQR 85,113–193,792). This pattern of decreasing ApB concentration at the third measurement was also observed, as mentioned above, in the whole cohort of patients, but did not reach statistical significance. Furthermore, using a multivariate binary logistic regression model (backward stepwise method) including other variables such as age, dyslipidemia, neurological evolution, previous stroke, functional dependence, and 3-month mortality, we found that CNS-ApB concentrations at baseline (< 9 h post-stroke) together with dyslipidemia and mortality were independent predictors of penumbra failure (Table 2). We then applied ROC analysis using data from all stroke patients included in this study (*n* = 71) as a discovery cohort to estimate the optimal cut-off value of plasma CNS-ApB levels at baseline for predicting recruitment or death of more than 20% of penumbra tissue. This revealed that at a concentration of 42,475 CNS-ApBs per 50 µl of plasma on the first determination, with an area under the curve of 0.73, the sensitivity and specificity of the measurement were 0.784 and 0.618, respectively (Fig. 3c).

**Table 2:** Linear regression analyses

Dependent variable	Independent variable	OR	95% CI	*p* value	Nagelkerke *R*^2^
Penumbra failure	Dyslipidemia	9.12872	2.52–42.1	0.001	0.34
	3-month mortality	16.83434	1.95–401.2	0.025	
	CNS-ApB levels (baseline)	1.00002	1.00001–1.00004	0.001	
Functional dependence at 3 m	Age	1.32947	1.11–1.80	0.013	0.72
	Baseline NIHSS	1.29601	1.11–1.62	0.004	
	Neurological evolution	38.0488	4.67–1441.6	0.009	
	CNS-ApB levels (baseline)	1.00003	1.00001–1.00006	0.02	

Penumbra failure: recruitment or death of more than 20% of penumbra tissue. CNS-ApB levels (baseline): concentration of apoptotic bodies from neurons and glial cells in the first determination (< 9 h from stroke onset). Neurological evolution: progressive, stable, regressive

**Fig. 4:**
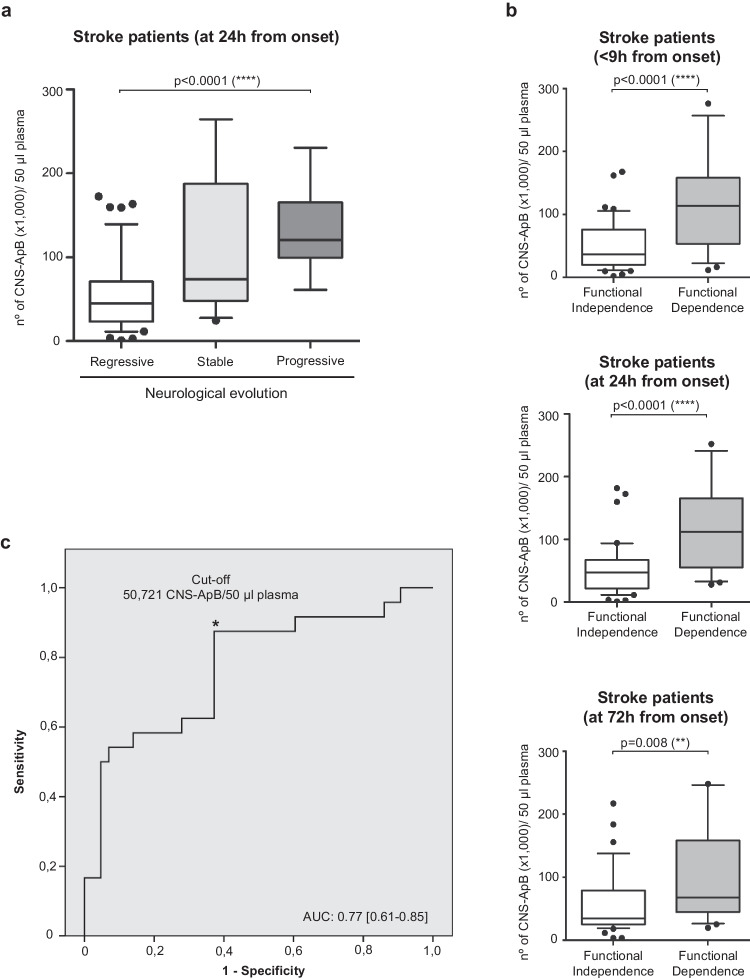
Associations between apoptotic bodies derived from brain tissue cells and neurological evolution and functional outcome. **a** Box plots represent the median and 95% CI of plasma apoptotic bodies (ApBs) concentrations derived from neurons, astrocytes, oligodendrocytes, and microglia/macrophages at 24 h according to the neurological evolution of ischemic stroke patients over the first 96 h. **b** Plasma CNS-ApB levels at the three time points analyzed and their relationship with functional dependence at 3 m from stroke onset. Lines in boxes and error bars represent median and 95% CI, respectively. **c** Estimation of the cut-off value of the concentration of circulating CNS-ApB determined within the first 9 h after stroke on ROC curve analysis for predicting dependence status

We next investigated the potential of CNS-ApBs as a predictive marker of neurological outcome. Spearman correlation analysis showed that CNS-ApB levels at baseline, 24 h, and 72 h were moderately and positively correlated with the initial clinical severity of stroke as estimated by the NIHSS score. In relation to the clinical evolution of the stroke during the first 96 h, it is noteworthy that patients who had a progressive course had significantly higher plasma CNS-ApB levels at 24 h post-stroke than those who remained stable or improved (Fig. 4a). Regarding functional status, plasma CNS-ApB levels during the first 72 h after symptom onset were also associated with patients’ functional prognosis. We also detected a significant association between the three determinations and functional independence at 3 months; patients with a favorable outcome (mRS ≤  ≤ 2) had significantly lower levels of circulating CNS-ApBs at < 9 h and 24 h and 72 h post-stroke than those with severe disability at 3 m (Fig. 4b). When the analysis was performed with the presence of poor functional dependency at 3 months as the dependent variable, the multivariate study (binary logistic regression, backward stepwise method) identified the following independent predictors: age, baseline NIHSS score, neurological evolution and baseline CNS-ApB plasma levels (Table 2). Furthermore, ROC analysis revealed that a concentration of 50.721 CNS-ApB/50 µl plasma within 9 h post-stroke predicted functional dependence at 3 months with an area under the curve of 0.77, and a sensitivity and specificity of 0.875 and 0.605, respectively (Fig. 4c). Finally, we found no association between ApB concentration and hemorrhagic transformation or with the presence of vascular risk factors.

## Discussion

In recent years, there has been growing interest in the field of extracellular vesicles (EVs) from the central nervous system, particularly microvesicles and exosomes. However, apoptotic bodies, vesicles formed as a result of cell fragmentation during the apoptotic process, have often been overlooked in studies of circulating vesicles. In this context, our group has developed a minimally time-consuming and easy-to-use method for the isolation and quantification of these types of vesicles from patient blood samples. Thus, the study of apoptosis by measuring ApBs has allowed, for the first time in the field of cerebrovascular disease, to analyze in vivo apoptosis in real time using a non-invasive technique and to identify the cell lineage of brain tissue undergoing apoptosis as a result of an ischemic insult. Our results showed the presence of higher levels of circulating ApB in neurons and glia in the acute phase of ischemic stroke compared to healthy subjects, confirming that activation of apoptosis in brain tissue plays a fundamental role as a mechanism of cell death after regional cerebral ischemic insult, as suggested by previous studies [11–13, 18–24, 33]. Interestingly, our data also showed that the plasma concentration of CNS-ApBs correlates with the proportion of penumbra that transforms into infarct tissue. Thus, the relationship found between circulating CNS-ApB levels, penumbra recruitment, and infarct growth supports the notion that apoptosis is a major mechanism involved in the death of ischemic penumbra tissue. In addition, neuronal and glial ApB levels in blood remained elevated during the first 3 days following stroke, despite a decrease at 72 h, suggesting that the process of apoptotic programmed cell death may continue for days after stroke onset. These results are consistent with other studies suggesting that apoptosis is the major cause of the progressive decline in neurological function after ischemic insult, which severely affects quality of life [29]. Regulation of several key proteins in the apoptosis pathway can reduce the size of cerebral infarcts, as has been shown in animal models of transient focal ischemia [28, 34–36] and the detection of apoptotic bodies in peripheral blood may be a useful marker of this process in vivo.

Although the cerebral ischemic lesion model has traditionally been approached from a neurocentric perspective, focusing solely on neurons, the role of macro- and microglia in the pathogenesis of stroke is now well established [37]. We have detected ApBs from both neurons and glia in relatively similar concentrations to those expected given the proportion of these cells in cerebral tissue, with the exception of astrocytes [38]. Although one-third of CNS-ApBs during the first 72 h after stroke is due to programmed astrocyte death, we expected to find a greater relative difference, as astrocytes represent more than 50% of brain tissue [38]. It is possible that this result can be explained by the resistance of astrocytes to ischemia, as they are capable of storing glycogen and possess robust antioxidant machinery [39].

We consider it a priority for further research to develop a therapeutic line focused on the mechanisms of blocking apoptosis, complementary to the revascularization techniques that are currently the mainstay of ischemic stroke treatment. In this context, the study of apoptosis by measuring plasma neuron- and glia-derived ApBs, as demonstrated in the present work, could represent an important advance in translational research in cerebrovascular pathology and other neurodegenerative diseases. It could also be used to monitor the response to various therapeutic options in cerebral ischemia, as well as for prognostic purposes and patient assessment.

Our study has several limitations, mainly related to imaging techniques. CTP was used to calculate the area of the ischemic penumbra. Ideally, infarct growth should be calculated at the expense of the progression of brain tissue at risk of irreversible death in the whole brain. However, this growth may have been underestimated in our study because the CT equipment used only allowed analysis of a maximum area of 4 cm along the craniocaudal axis. In addition, due to the size of the patient cohort (*n* = 71), the optimal CNS-ApB cut-off values obtained for penumbra failure and functional dependence were not validated in an independent group of stroke patients.

In summary, this study supports the hypothesis that activation of apoptotic processes in the ischemic penumbra during cerebral infarction contributes to tissue death in this area after an ischemic insult and may also explain the progressive evolution in some stroke patients. It seems clear that CNS-ApBs are plasma biomarkers of in vivo apoptosis in cerebral ischemia and have clinical utility as an early predictor of cerebral infarct growth, early neurological deterioration, and functional outcome.

## Data Availability

No datasets were generated or analysed during the current study.
